# In this month’s Bulletin

**DOI:** 10.2471/BLT.18.000718

**Published:** 2018-07-01

In the editorial section, Beverley M Essue et al. (442) discuss policies designed to address the high cost of care for chronic kidney disease in India. Yvan Hutin et al. (443) describe milestones in the reduction of hepatitis B infections. 

Sophie Cousins (446–447) reports on cholera vaccination programmes in crisis settings. Yoshitake Yokokura tells Fiona Fleck (448–449) how Japan proposes to direct primary health care services towards the needs of an ageing population. 

## Burkina Faso

### Changing paternal roles

Marina Daniele et al. (450–461) study the inclusion of male partners in maternity care.

## Cambodia

### How much does a health system cost?

Catherine Barker Cantelmo et al. (462–470) estimate the costs of a 5-year national health plan.

## China

### Establishing the impact of tobacco tax 

Mark Goodchild & Rong Zheng (506–512) assess the impact of a recent tax increase.

## Global

### Cash interventions and pulmonary tuberculosis

Aaron Richterman et al. (471–483) collect the evidence of effects on clinical outcomes.

### Hepatitis B prevalence

Kate Whitford et al. (484–497) review long-term impacts of infant immunization programmes. 

### Tracking harms in primary care

Jennifer Cooper et al. (498–505) propose a classification for patient-safety incidents.

